# Supplementary material for: A novel nonsense variant in SUPT20H gene associated with Rheumatoid Arthritis identified by Whole Exome Sequencing of multiplex families
Source: PLoS One. 2019 Mar 7;14(3):e0213387. doi: 10.1371/journal.pone.0213387 (PMC6405192; doi:10.1371/journal.pone.0213387)
Supplement: S1 Table — a Number of index cases / Number of index cases with data b previous and/or actual tobacco exposure (smokers and ex-smokers). RF: Rheumatoid Factor. ACPA: Anti-Cyclic Citrullinated Peptide Antibodies. (DOCX) [file pone.0213387.s004.docx]

|  | Number^a^ | Percentage |
| --- | --- | --- |
| Female | 168/188 | 89.36 % |
| Age at onset less than 40 years | 133/188 | 70.74 % |
| RF positive | 140/188 | 74.46 % |
| ACPA positive | 135/175 | 77.14 % |
| Presence of Erosion | 156/188 | 82.98 % |
| Presence of Nodules | 47/181 | 25.97 % |
| Tobacco Exposure^b^ | 88/167 | 52.70 % |
| Carrying at least one HLA-DRB1 shared epitope allele | 144/188 | 76.59 % |
